# Audit of Compliance With Section 3 Mental Health Act Capacity Assessment and Three-Month Reassessment in a Psychiatric Inpatient Unit

**DOI:** 10.1192/bjo.2026.11503

**Published:** 2026-06-30

**Authors:** Kaushiki Tripathi, Aslihan Arslan Argin, Beena Rajkumar

**Affiliations:** 1 South London and Maudsley NHS Foundation Trust (SLaM), United Kingdom.; 2 Lincolnshire Partnership NHS Foundation Trust (LPFT), United Kingdom

## Abstract

**Aims:**

Under the Mental Health Act (MHA) 1983, patients detained under Section 3 must have their capacity to consent to treatment assessed on admission and reassessed at the three-month point. Failure to complete these assessments risks breaching patient rights, including those protected under the Human Rights Act, and undermines lawful and person-centred clinical practice.

Aims were (1) To determine compliance with documentation of capacity assessment at admission and at the three-month point for Section 3 inpatients; (2) to identify practical improvements to enhance compliance.

**Methods:**

A retrospective audit was conducted on 13 inpatients detained under Section 3 on Vales Ward, Discovery House (LPFT). Admission dates and capacity-assessment records were extracted from the RiO electronic patient record for the period December 2019–November 2022. The primary outcomes were the presence or absence of documented capacity assessment at admission and at the three-month review.

**Results:**

Capacity assessment at admission was documented in 46.2%(6/13) and not documented in 53.8%(7/13).

Three-month reassessment was documented in 7.7%(1/13) and not documented in 92.3%(12/13).

**Conclusion:**

Compliance with MHA Section 3 statutory requirements for capacity assessment was poor, particularly at the three-month review. We recommend: (1) implementing automated RiO reminders to clinical and MHA administration teams at −1 month, −1 week, and −1 day before the three-month point; (2) delivering targeted staff education on the legal and clinical significance of capacity reassessment; and (3) providing patient information leaflets regarding consent and MHA rights. A reauditis planned to evaluate the impact of these interventions.

